# A new species of *Coomaniella* (Coleoptera, Buprestidae, Coomaniellini) from Guangxi, China, with new distributional records and biological observations

**DOI:** 10.3897/zookeys.110.59530

**Published:** 2021-01-13

**Authors:** Hai-Tian Song

**Affiliations:** 1 Fujian Academy of Forestry Sciences, Fuzhou, Fujian, 350012, China Fujian Academy of Forestry Sciences Fuzhou China

**Keywords:** Host plant, new distributional records, new species, taxonomy

## Abstract

A new species Coomaniella (Coomaniella) dentata**sp. nov.** from Guangxi Province, China, is described and placed in the C. (C.) chinensis species-group. The variability is discussed, and a new adult host plant is recorded. New distributional records are given for *C. biformissima* Jendek & Kalashian, 1999 and *C. lingafelteri* Jendek & Pham, 2013.

## Introduction

Bourgoin (1924) proposed the genus *Coomaniella* for two species. Years later, Bílý (1974) established a new tribe, Coomaniellini, for it and discussed its systematic position in the subfamily Buprestinae. Jendek and Kalashian (1999) revised the genus, dividing it into two new subgenera and eight species-groups, described new species, and provided keys. More new species were found, species-groups were redefined, and the distribution and biology were summarized in the last couple of decades (Jendek 2002, 2005; Jendek and Pham 2013). Currently, the genus contains three subgenera and 31 species. Bílý and Volkovitsh (2015) discussed the status of the tribe Coomaniellini and described the larval stage of Coomaniella (Coomaniella) purpurascens Baudon, 1966.

In this paper, a new species belonging to the C. (C.) chinensis species-group is described. New distributional records for two species from Guangxi province, China, are given, and a new adult host plant is recorded.

## Materials and methods

Abbreviations for collections in this study are:

**CHTS** Collection of Hai-Tian Song, Fuzhou, China;

**FAFS** Fujian Academy of Forestry Sciences, Fuzhou, China.

Whole specimens were photographed using a Keyence VHX-5000 digital microscope with the Keyence VH-Z20R zoom lens (20–200×). The feeding behavior was photographed using Canon 5D4 digital camera with the Canon 100 mm f/2.8 macro USM. The host plant and photographs in nature were taken using a Huawei smartphone. The images were processed and combined into figures using Adobe Photoshop CC 2019.

## Taxonomy

### Coomaniella dentata sp. nov.

Taxon classificationAnimaliaColeopteraBuprestidae

0D21403B-EDE7-5075-8006-047DE7974631

http://zoobank.org/E98B9837-24F7-489B-BC60-CD0C3E3F2D47

Figures 1 , 2

#### Type specimens.

***Holotype*** ♂ (FAFS): China • Wuzhi Mountains, Yao Autonomous County of Jinxiu, Laibin City, Guangxi; 23°54'N, 110°9'E, alt. 700 m; 14–15.V.2020; Chun-Fu Feng leg. ***Paratypes*** 12 ♂♂, 17 ♀♀ (CHTS): China • same collection data as for holotype • 3 ♂♂, 4 ♀♀ (CHTS); same collection data as for preceding; 25–26.V.2020 • 1 ♂ (CHTS); same collection data as for preceding; 11.IV.2020.

#### Description of the holotype.

***Size***: body length 6.55 mm, width 2.14 mm.

**Figure 1.**
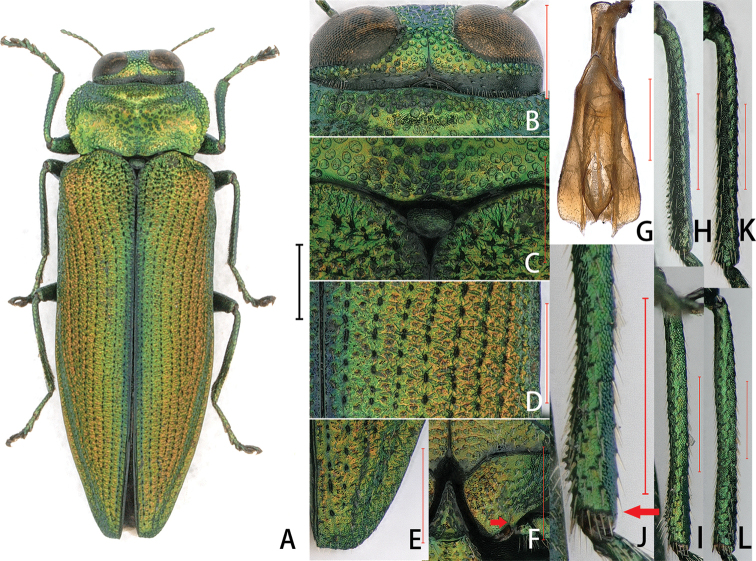
Coomaniella (Coomaniella) dentata sp. nov. **A–J** holotype: **A** habitus **B** head **C** scutellum **D** elytral surface **E** elytral apex **F** metacoxal plate with spine **G** aedeagus **H** male mesotibia, dorsal view **I** male metatibia, ventral view **J** denticles on male metatibia, ventral view **K, L** paratype: **K** female mesotibia, dorsal view **L** female metatibia, ventral view. Scale bars: 1 mm (**A**); 0. 5 mm (**B–L**).

***Body***: suboval, elongate; head, pronotum, legs, ventral side, appendices, and antenna golden-green; elytra color horizontal gradient, golden-blue near suture, golden-green in central and golden-orange outsides (Fig. 1A). Dorsal side with short, sparse, pale pubescence. ***Head***: vertex roughly punctate, in narrowest part between the eyes reduced to six rows of punctures (Fig. 1B); antennae short, scarcely reaching to anterior pronotal corners, markedly shorter than length of pronotum. ***Pronotum***: strongly transverse, 1.92× as wide as long, widest in the middle, sides deeply arcuate; anterior lobe existing, anterior margin narrower than posterior; disk with obvious, deep impressions (Fig. 1A). ***Scutellum***: very small, subpentagonal with corners very obtuse; impressed on disk (Fig. 1C). ***Elytra***: about 2.43× as long as wide; rugoso-punctate, striae well marked; covered with asperate, dense sculptures and obvious longitudinal sequential punctures (Fig. 1D); elytral apices simplex, subtruncate with subangulate angle at sutural margin and without spines (Fig. 1E). ***Ventral side***: inner posterior angle of metacoxal plates subangulate, with a big spine beside (Fig. 1F). Sternal carina flanked with groove. ***Legs***: tibia with apical spur and many hairs. Mesotibia and metatibia in distal half of inner margin with denticles (Fig. 1H–J); tarsal shapes unchanged; tarsus distinctly longer than half of corresponding tibia; tarsomere 1 shorted than next three tarsomeres combined. ***Aedeagus***: membranous, with much wider parameres (Fig. 1G).

#### Sexual dimorphism.

Male eyes separated on vertex in narrowest part by six to eight rows of punctures, while female by nine or 10. Protarsomere 1 obviously lengthened in male; mesotibia and metatibia with denticles in distal half of inner margin in male (Fig. 1H–J) but no denticles in female (Fig. 1K, L). Antennal length similar in both sexes.

#### Variability.

**Figure 2.**
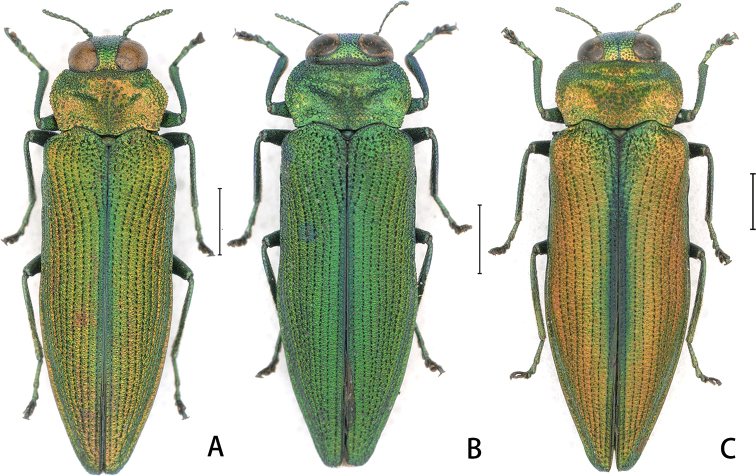
Variability of Coomaniella (Coomaniella) dentata sp. nov. **A** male, pronotal variety **B** male, green variety **C** female, orange variety. Scale bar: 1 mm.

Body 3.05–3.40 × as long as wide. Pronotum sometimes variable in shape (Fig. 2A). Color variable and both sexes have green (Fig. 2B) and orange forms (Fig. 2C). Aspect ratio of scutellum variable. Elytral apices from subarcuate to subtruncate, subangulate angle at sutural margin sometimes weakened or even absent (Fig. 2A–C). Body length of sexes overlap: male 5.78–7.53 mm and female 6.80–8.46 mm.

#### Discussion.

This new species presents several diagnostic characters which allow it to be easily separated from other species: 1) mesotibia and metatibia in males with denticles, 2) elytral apices without spines, 3) metacoxal plates with a big spine beside the inner posterior angle in both sexes, 4) elytral surface with obvious longitudinal punctures, and 5) the narrowest part between the eyes on each sex with more rows of punctures. The first two characters are reported for the first time in genus *Coomaniella*, adding to the structural diversity of the genus. The new species is classified in the subgenus Coomaniella and belongs to C. (C.) chinensis species-group.

#### Etymology.

The name *dentata* is for the denticles on male tibia.

#### Host plant.

**Figure 3.**
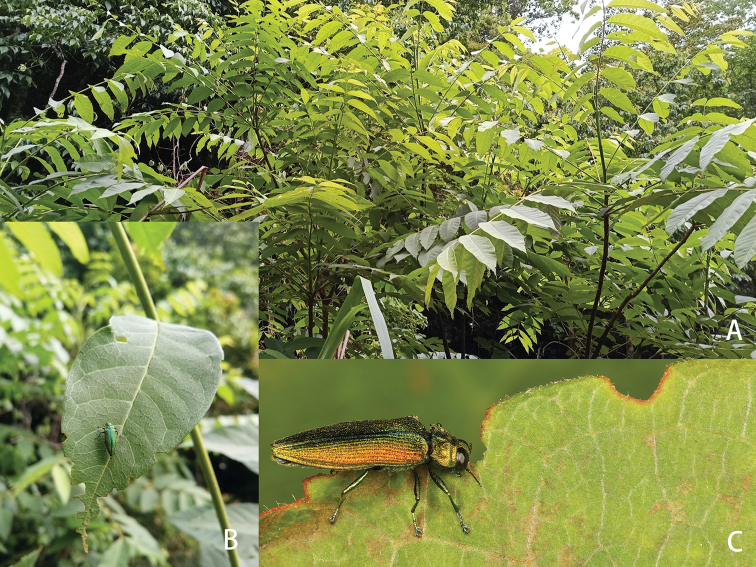
Host plant and habits of Coomaniella (Coomaniella) dentata sp. nov. **A***Toona* sp. in the wild **B** marginal feeding of the leaf of *Toona* sp. by *C. dentata***C** feeding habits.

*Toona* sp. (Fig. 3A, B), determined by Prof. Xin-Hua Li (Nanjing Agricultural University), is a newly reported genus of adult host plant for the genus *Coomaniella*. *Toona* sp. belongs to the family Meliaceae, of which another species, *Chukrasia tabularis*, has been recorded as a host plant by Jendek (2002).

#### Remarks.

All specimens of *C. dentata* sp. nov. were collected during hot sunny days on both the top and bottom surfaces of the leaves of *Toona* sp. (Fig. 3A). Margin feeding on the leaves was observed and was sometimes quite obvious (Fig. 3B). Laboratory breeding showed that *C. dentata* sp. nov. could indeed feed on this plant (Fig. 3C). No larval galleries were found.

##### From the same host plant, two other species were collected

### Coomaniella (Coomaniella) biformissima

Taxon classificationAnimaliaColeopteraBuprestidae

Jendek & Kalashian, 1999

F4ABB10E-4820-5B22-B0DD-60CE85C3C5AE

#### Material examined.

7 ♂♂, 5 ♀♀(CHTS): China Wuzhi Mountains, Yao Autonomous County of Jinxiu, Laibin City, Guangxi; 23°54'N, 110°9'E, alt. 700 m; 14–29.V.2020; Chun-Fu Feng leg. New country record.

### Coomaniella (Coomaniella) lingafelteri

Taxon classificationAnimaliaColeopteraBuprestidae

Jendek & Pham, 2013

179181AE-370E-5586-A5E1-BD18DF2B457D

#### Material examined.

8 ♂♂, 7 ♀♀(CHTS): China, Wuzhi Mountains, Yao Autonomous County of Jinxiu, Laibin City, Guangxi; 23°54'N, 110°9'E, alt. 700 m; 14–29.V.2020; Chun-Fu Feng leg. New country record.

## Supplementary Material

XML Treatment for Coomaniella dentata

XML Treatment for Coomaniella (Coomaniella) biformissima

XML Treatment for Coomaniella (Coomaniella) lingafelteri
